# The Environmental Profile of Ecuadorian Export Banana: A Life Cycle Assessment

**DOI:** 10.3390/foods11203288

**Published:** 2022-10-20

**Authors:** Kevin Veliz, Leticia Chico-Santamarta, Angel D. Ramirez

**Affiliations:** 1 Facultad de Ingeniería en Mecánica y Ciencias de la Producción, Escuela Superior Politécnica del Litoral, ESPOL, Campus Gustavo Galindo, Km. 30.5 Vía Perimetral, Guayaquil P.O. Box 09-01-5863, Ecuador; 2 International Department, Harper Adams University, Newport TF10 8NB, UK

**Keywords:** banana, LCA, carbon footprint, greenhouse gas emissions, environmental footprint, export, transport, Ecuador

## Abstract

Ecuador is one of the largest banana exporters in the world. This sector generates wealth and employment in the country. Life cycle method tools support finding critical points and improvement measures in systems. In this study, the Ecuadorian banana is evaluated through life cycle assessment (LCA), including agriculture, packaging, transfer to the Port of Guayaquil, and transport to a foreign port. OpenLCA software was used, applying the Recipe Midpoint (H) V1.13 impact evaluation method and using primary data collected from a local producer and secondary data from Ecoinvent 3.6 databases, Agribalyse 3.0.1, and the literature. Functional units were established at three levels: “1 ton of Banana at-the-farm-gate”; “1 ton of Banana at-the-packaging-stage-gate”; and “1 ton of Banana at-the-port-of-destination”. The impact categories evaluated are climate change (GWP100), fossil depletion (FDP), freshwater eutrophication (FEP), marine eutrophication (MEP), ozone layer depletion (ODPinf), particulate matter formation (PMFP), formation of photochemical oxidants (POFP), and terrestrial acidification (TAP100). The carbon footprint (GWP100) of “Banana at-the-farm-gate”, “Banana at-the-packaging-stage-gate”, and “Banana at-the-foreign-port” ranged from 194 to 220, 342 to 352, and 615.41 to 625.44 kg CO_2_-Eq/Ton banana, respectively. Hotspots of the system are the fertilizer field emissions, cardboard packaging, rachis disposal, and maritime transport. Improvement measures should focus on reducing the amount of fertilizers and developing circular alternatives for residual biomass valorization.

## 1. Introduction

Bananas are among the most important crops in agricultural production worldwide; most of the world’s crops are informal and grown by small informal farmers [1]. Accessible estimates indicate that the global average banana production increased by 68% from 2000 to 2019 [2]. World banana exports in 2020 reached a record 22.2 million metric tons, an increase of 1.7% compared with 2019; this is caused by the increase in supply in Ecuador, Costa Rica, and Colombia [3]. For the year 2021, estimates indicate that world banana exports decreased by 7%. In Ecuador, the estimated decrease was 4% of exports, which is approximately 6.8 million tons [4].

Countries involved in agricultural production are affected by climate change and farmers’ attempts to adapt to extreme weather conditions [5]. This includes increasing the use of irrigation, the greater use of fertilizers, and agricultural intensification. The expansion of land for agricultural practices may lead to a loss of biodiversity, reducing taxonomic richness, and approaching a point where fertilizers would not improve production results [6].

Cavendish banana exports from Ecuador represent more than 30% of world exports, and its production represents 40% of world production [7]. The banana sector is a pillar of the country’s economic development, creating employment, especially in the coastal region, and producing export food, and is a product of significant consumption in the foreign markets [8]. In 2018, bananas represented 30% of the economic income of agricultural exports in Ecuador and in 2020 they represented one of the three products with the highest sales abroad, along with coffee and cocoa [9]. Between 2019 and 2020, the economic income generated by the export of bananas and plantains increased from USD 3295.2 million to USD 3669.0 million (11.3%) [10].

**Life cycle assessment (LCA) is a methodological framework for evaluating the environmental impacts of products and services standardized by ISO** [11]. LCA can be used to assess different impact categories [12]. Table 1 includes the description of various studies, the limits of the systems, the scope of the geographic systems, the functional unit, and the impact categories evaluated. Most studies have mainly focused on calculating the climate change indicator rather than on a full LCA approach. There are cases of other countries, such as Brazil [13], Uganda [14], Costa Rica [15], and Switzerland [16], where other impact categories have been evaluated, as shown in Table 1. In Ecuador, there are previous banana LCA studies [12,17,18,19] focused on determining the carbon footprint of Cavendish bananas. There is no previous LCA study that includes other impact indicators that are important in agricultural systems. Thus, there is a need for the development of complete LCA studies of this fruit of extreme relevance for Ecuador.

Life cycle assessment has been used to describe the sustainability of food production around the world, but these types of studies are not frequently used for Ecuadorian conditions. Regarding biosystems, the LCA methodology has been used in Ecuador for agricultural and food products [22,23,24,25] and bio-based materials and biofuels [26,27,28,29,30].

The aim of this study is to evaluate the environmental performance of a fruit of the genus Musa, from the AAA group “Banano Cavendish” produced in Ecuador, using the LCA methodological framework for a case study in order to: (i) identify hotspots and opportunities to mitigate environmental impact and (ii) compare with available results of the carbon footprint of previous studies of the Ecuadorian banana.

## 2. Materials and Methods

The International Standard ISO 14040: 2006 used for this work specifies the four phases that make up a typical LCA, starting with the definition of the objective and scope, followed by an inventory analysis, continuing with the impact assessment, and ending with the interpretation of the results [11].

### 2.1. Scope Definition

#### 2.1.1. Functional Unit

Three functional units are used: “1 ton of banana at-the-farm-gate”; “1 ton of banana at-the-packaging-plant-gate”; “1 ton of banana at-the-port-of-destination”.

#### 2.1.2. System Boundaries

The temporal limits of the system correspond to data recorded between 1 January 2019 and 31 December 2020. The geographical limits range from the agricultural production located in the city of Quevedo in the province of Los Ríos (Ecuador) to the point of delivery in a foreign port.

**Figure 1:**
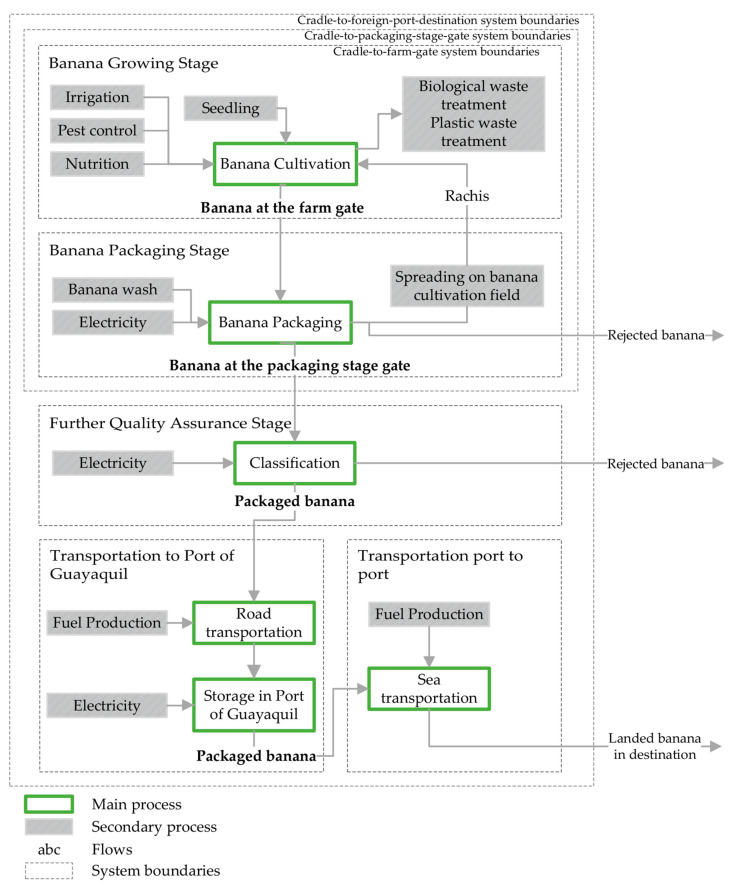
System boundaries at cradle-to-farm-gate, cradle-to-packaging-stage-gate, and cradle-to-foreign-port-destination for the studied system.

Figure 1 shows the three system boundaries used (i.e., cradle-to-farm gate, cradle-to-packing gate, and cradle-to-foreign port of destination) and the stage boundaries; these include main processes and flows.

The information on inputs and outputs, for the period studied at the levels of “Banana at-the-farm-gate” and “Banana at-the-packaging-stage-gate”, is available in Supplementary Tables S1 and S2, respectively, in the Supplementary Information.

#### 2.1.3. System Description

##### Banana Growing Stage

Banana cultivation (Musa × paradisiaca AAA) commonly involves cultivation activities such as irrigation, fertilization, shoring, defoliation, and pest and disease control [31], in addition to the post-cultivation phases that include harvesting and packaging. The Cavendish banana represents the most common banana for commercial exports, due to the absence of seeds and the content of potassium (i.e., 400 milligrams per 100 g of fresh fruit) [32].

The fertilization of the field is done by manual techniques. For these types of crops, it is common to use nitrogen as an input for soil and fruit nutrition. Nitrogen fertilizers used on the farm for this work during the years 2019 and 2020 were urea, ammonium nitrate, ammonium phosphate, ammonium sulphate, and NPK fertilizers.

Water application was executed through an irrigation system composed of a drainage network with a main channel that distributes the water into secondary channels to be used in a spraying foliar irrigation system. The system works with a diesel combustion engine.

Pest control was done by spraying at foliar and subfoliar levels with manual and petrol pumps. Another method, used to a lesser extent, is an aerial spraying plane, which is used depending on the time of the year. In the winter, due to the frequency of the rain, this method is not frequently used. Furthermore, spraying with the plane is mainly to control the Black Sigatoka (*Mycosphaerella fijiensis*), and the frequency of the application depends on the sanitary controls. Pesticides used during this period include prochloraz, diquat, paraquat, fenpropidine, glyphosate, glufosinate-ammonium, thiamethoxam, imidacloprid, chlorothalonil, manconzeb, pyrimethanil, terbufos, metiram, azoxystrobin, and spiroxamine.

Agricultural plastics impregnated with insecticide are used to protect bananas to keep the fruit from external dangers (e.g., insects, garbage, wind) and to avoid contamination and damage to the banana. The characteristics of the plant and the weight of the bunches can cause them to fall as a result of their weight and, thus, damage the fruit. Plastic straps or wooden posts are used to prevent these accidents.

##### Banana Packaging Stage

Packaging is defined as the stage in which the cultivation phase ends. The fruit is prepared to be packaged together with other special components that will serve as a barrier against unwanted external factors and as a permeable material that helps maintain the balance of the interior environment, optimizing the metabolic processes of the fruit [33]. Cardboard boxes, dividers, and plastic bags are the most common packaging materials for bananas, and their design, size, and materials are chosen to ensure product quality [34].

In this work, the production of cardboard was taken into account, including the stages of cutting, folding, and printing with a gravure machine and including the impacts due to the use of inks and glues, in addition to electricity consumption. The production of separators and plastic bags used for packing bananas was also taken into account. When using plastic bags, a vacuum cleaner is also used to extract the air in order to lengthen the ripening time of the fruit, preserving it better during long journeys.

The rachis is a residue of this stage. In this study, the rachis management is also included as a part of the packaging stage. Three possible scenarios were proposed at the level “Banana at-the-packaging-stage-gate”(Table 2). The first scenario (RM0) considered nitrogen emissions and treatment. The second scenario (RM1) considers only the nitrogen emissions. The third scenario (RM2) does not include the burden of the rachis.

In the packaging stage, it is also verified that the banana complies with all the parameters requested by each buyer. The fruit that does not meet the necessary qualities is not packaged, requiring a greater quantity of fruit to complete 1 ton of bananas. In this case, a rejection percentage value of 5% was considered, according to the records reviewed on the farm and recommendations from the field staff.

##### Further Quality Assurance Stage

Before being received at the Port of Guayaquil, on certain occasions, an additional quality check is performed, where a random box is opened to check that the product and the packaging meet all the required provisions. This is only carried out at the request of the exporting company.

##### Transport to the Port of Guayaquil

The transport stage to the departure port starts after the packaging stage when the fruit is stowed in vehicles for transport, generally in trucks and containers, and where the fruit is temporarily stored in bulk and pallets. In Ecuador, the main destinations are the Libertador Simón Bolívar Maritime Port in the city of Guayaquil and the Bolívar Port in the city of Machala. For this case the Port of Guayaquil is used.

##### Transport to Foreign Port Destination

The stage of transport to the foreign port of destination begins after the containers arrive at the departure port and are stowed on the container ships. Currently, the main ports of destination for Ecuadorian bananas are located in the countries of the United States, Holland, Turkey, Russia, and China. In this study, to identify and compare the contributions of this stage from departure from the port in Ecuador to arrival at the foreign port, two destination points were used: the Port of San Diego in the United States (5917 km) and the Port of Rotterdam in Holland (10,820 km).

### 2.2. Life Cycle Inventory Analysis

#### 2.2.1. Data on Agricultural Inputs

##### Direct Agricultural Inputs (Foreground System)

To prepare the inventory, information necessary to quantify the flow of materials and emissions from the system was requested. The case study inventory was carried out on a conventional farm of 200 ha, with the input data of products such as seedlings, fertilizers, pesticides, water consumption, fuel consumption, electricity consumption, cleaning products, plastics, and packaging material obtained from farm records and interviews with the technical and administrative personnel of the farm.

The output data for the banana production and the production of biological waste were obtained through records and calculations based on an average percentage. The amount of plastic packaging waste was calculated based on the average for this type of product and assuming polyethylene as the packaging material.

Tables that present the input and output data, at the levels of “Banana at-the-farm-gate” and “Banana at-the-packaging-stage-gate”, are available in the Supplementary Information.

##### Background Processes

The study includes processes for the production of agricultural inputs such as fertilizers, pesticides, and diesel and materials for irrigation, protection, and packaging. Background processes of life cycle inventories were incorporated from Ecoinvent 3.6. and Agribalyse 3.0.1. databases [35,36,37]. Electricity was derived from Ramirez et al., [38,39]. The management of rachis has been modeled as an open dump using a process of Ecoinvent [36].

#### 2.2.2. Determination of Field Emissions

##### Emissions from the Application of Fertilizers and Agricultural Lime

For the emissions of agricultural fertilizers, the IPCC emission factors were used to establish the CO_2_ (carbon dioxide) emissions from the application of lime and urea [40]. In the case of N_2_O (nitrous oxide), emissions from the use of nitrogen fertilizers, for the purpose of this study, it was modified and emission factors for tropical climates were used [41]. NO_x_ (nitrogen oxides) emissions were calculated using the Ecoinvent emission factors proposed by [42] for agricultural production systems. NH_3_ (ammonia) emissions were determined using the Carnegie Mellon University (CMU) fertilizer emission factors published by the Environmental Protection Agency (EPA) [43]. The calculations to determine the leachates of NO_3_ (nitrates) were determined according to the methodology published by [44] for direct emissions from the field and the farm. For PO_4_ (phosphate) emissions, the method proposed by [45] was used. Emissions produced from agricultural residues, such as rachis, that are decomposed in the field were calculated considering the nitrogen content of 1,9% [46].

##### Emissions Associated with the Application of Pesticides

The emissions produced by the application of all the pesticides used in the three crops were calculated using the Analytica software as a visual tool for the calculation models [47]. The PestLCI 2.0 model, developed by the Technical University of Denmark (TUD), was used to estimate the pesticide emissions to air, surface, and groundwater [48].

### 2.3. Life cycle Impact Assessment

#### Impact Assessment Methodology

The calculation of the impact category indicator results was performed using the Recipe Midpoint (H) V1.13 impact evaluation method, which is one of the most used methods in current LCA studies. The impact categories analyzed were: global warming potential (GWP100), fossil depletion potential (FDP), freshwater eutrophication potential (FEP), marine eutrophication potential (MEP), ozone depletion potential (ODPinf), particulate material formation potential (PMFP), photochemical oxidants formation potential (POPF), terrestrial acidification potential (TAP100). OpenLCA software was used to evaluate the environmental impacts of the life cycle assessment.

## 3. Results and Discussion

### 3.1. Life Cycle Inventory Analysis

Supplementary Table S1 shows the inputs and outputs for the “Banana Growing Stage”. Supplementary Table S2 shows the inputs and outputs for the “Banana Packaging Stage”, available in the Supplementary Information.

### 3.2. Life Cycle Impact Assessment

#### 3.2.1. Banana at-the-Farm-Gate

**Figure 2:**
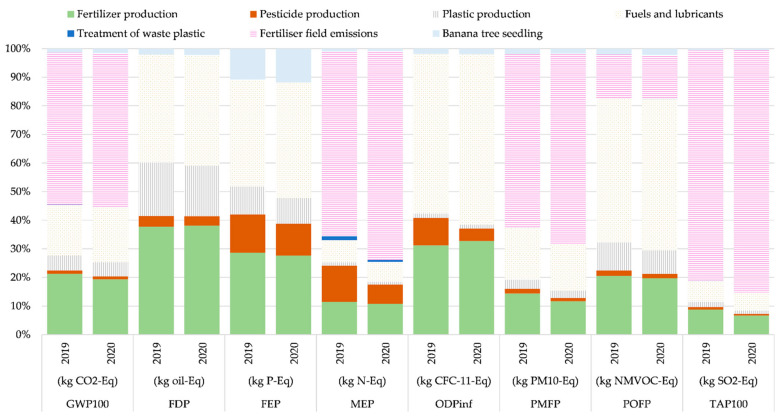
Contribution analysis of processes for the flow of “Banana at-the-farm-gate”, classified by impact category for the periods 2019 and 2020.

Figure 2 shows the percentage contributions of inputs and outputs to each environmental impact category for the banana growing stage.

The flows involved in this stage were grouped by type (i.e., fertilizer production, pesticide production, plastic production, fuels and lubricants, plastic waste treatment, fertilizer field emissions, banana tree seedling). The flow “fertilizer field emissions” is the highest contributor in the categories GWP100 (52–53%), MEP (62–71%), PMFP (59–62%), and TAP100 (80–82%). This is in agreement with previous studies [12,17], where also the highest emission is associated with the application of a nitrogen fertilizer, such as ammonium nitrate, diammonium phosphate, or urea. For MEP, the highest contribution is associated with aminopyridine and water pump operation. For PMFP and TAP100, the most important processes are the water pump operation, ammonium nitrate, and urea.

The production of “fuels and lubricants” is the highest contributor for FEP (37–40%), ODPinf (56–60%), and POPF (50–53%). For FEP and ODPinf, the highest contribution is associated with the water pump operation. For FDP, the flows of “fuels and lubricants” and “fertilizer production” contributed equally (37–38%). This is mainly associated with the water pump operation and the production of urea.

Table 3 shows the contributions of the flows to each impact category, in the period studied, for 1 ton “Banana at-the-farm-gate”.

For the GWP100 category, the highest contributors were fertilizer field emissions, followed by fertilizer production and the production of fuels and lubricants. In both years, these three flows represented more than 90% of CO_2_-eq emissions. In the period studied, the main nitrogenous fertilizers used were ammonium nitrate, diammonium phosphate, and urea, representing the highest impact in four categories. For GWP100, the emission of dinitrogen monoxide represents 53%. The emissions of ammonia and nitrogen oxide represent 65%, 61%, and 81%, for the MEP, PMFP, and TAP100 categories, respectively.

The contributions of “fuel and lubricants” are associated with diesel as the fuel for the operation of the water pump, the use of kerosene for aerial fumigation operations, the use of gasoline for spraying with motorized pumps, and the use of lubricating oils for internal combustion engines. The production of “fuels and lubricants” is the highest contributor for FEP, ODPinf, and POPF. For the categories FEP and ODPinf, the high emission is associated with the water pump operation for the years 2019 and 2020.

The production of “fuels and lubricants” and “fertilizer production” contributed equally in the category FDP. The contribution is associated with the water pump operation and the urea for the years 2019 and 2020.

#### 3.2.2. Banana at-the-Packaging-Stage-Gate

**Figure 3:**
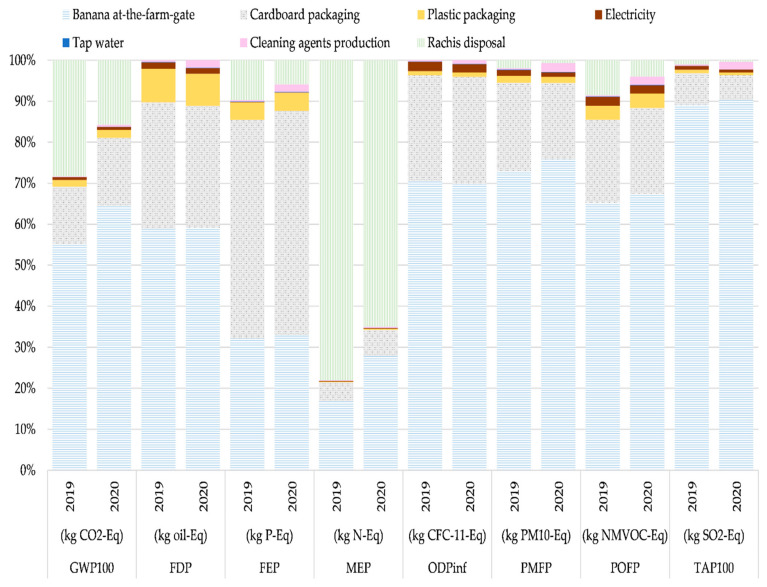
Contribution analysis of processes for the flow of “Banana at-the-packaging-stage-gate”, classified by impact category for the periods 2019 and 2020.

Figure 3 shows the percentage contributions of inputs and outputs to each environmental impact category for the banana packaging stage.

The flows involved in this stage were grouped by type (i.e., “Banana at-the-farm-gate”, cardboard packaging, plastic packaging, electricity supply, tap water, cleaning agent production, rachis disposal). The product flow of “Banana at-the-farm-gate” is the highest contributor in the impact categories GWP100 (56–66%), FDP (59–60%), ODPinf (71–72%), PMFP (74–77%), POFP (66–68%), and TAP100 (89–90%). The FEP category was an exception, where the production of cardboard packaging (53–54%) was the highest contributor. The MEP category was another exception, where the highest contributor was rachis disposal (64–77%).

The cardboard packaging is the second highest contributor in all categories: GWP100 (15–16%), FDP (30–31%), FEP (53–54%), MEP (5–7%), ODPinf (26%), PMFP (18–21%), POFP (20–21%), andTAP100 (7–8%). In the case of the GWP100 category, the majority of emissions is associated with cardboard box production (83–84%). This was compared with other studies, in the case of Roibas et al. [17] for the contributions from cardboard manufacturing (89%), in the case of Iriarte et al. [12] associated with cardboard boxes and kraft paper manufacturing (91%), and in one case from Brazil, Coltro and Karaski [13], associated with the cardboard used to pack the bananas (83%).

The “rachis disposal” is the third highest contributor in this stage in the categories GWP100 (16–28%), FEP (6–10%), MEP (65–78%), and POFP (4–8%). For the GWP100 category, the contribution is associated with the elemental flow of methane.

Table 4 shows the contributions of the flows to each impact category, in the period studied, for the 1 ton “Banana at-the-packaging-stage-gate level”.

The product flow “Banana at-the-farm-gate” is the highest contributor in most impact categories, GWP100, FDP, ODPinf, PMFP, POFP, and TAP100, for the years 2019 and 2020. In the FEP category, cardboard packaging was the one with the highest contribution; this contribution is associated with the production of electricity. In the MEP category, the highest contributor was rachis disposal for the years 2019 and 2020; the highest environmental burden is associated with the open dump disposal.

Cardboard packaging is the second highest contributor in the categories FDP, FEP, ODPinf, PMFP, POFP, and TAP100. For the FDP and ODPinf categories, the contribution is associated with petroleum and gas production. For the FEP, PMFP, POFP, and TAP100 categories, the contribution is associated with electricity production.

The flow “rachis disposal” is the third highest contributor in the categories GWP100, FEP, MEP, and POFP for the years 2019 and 2020. In the cases of GWP100 and POFP, the contribution is associated with the emissions of methane; for FEP, phosphate emissions to water are the main cause; for MEP, nitrogen emissions to water are the main causes.

Table 5 shows the impact category indicator results, in the period studied, for the flow of “Banana at-the-packaging-stage-gate” in the three different rachis management scenarios described in Table 2.

The scenarios RM1 and RM2 resulted in a reduction in almost all impact category indicator results in comparison with RM0. There are exceptions in the ODPinf and FDP categories, where there were no changes. For the GWP100 category, in the RM1 and RM2 scenarios, it was reduced by 9% and 28%, respectively. In the FEP category, in the RM1 it was reduced by 10% and in the RM2 it was not reduced. These differences are associated with the way in which the scenarios were developed. RM1 does not include nitrogen emissions from the rachis and RM2 does not include any environmental load associated with the rachis management. Currently, there is no information that indicates whether either RM0 or RM1 is more realistic.

#### 3.2.3. Banana at-the-Foreign-Port

**Figure 4:**
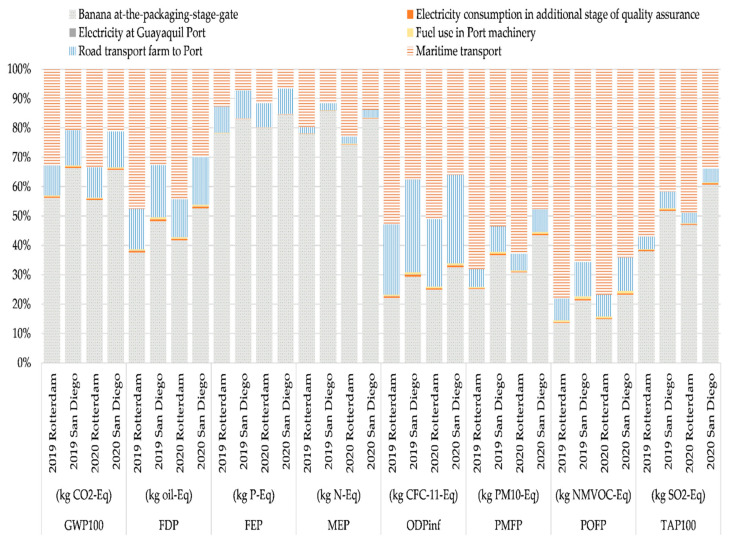
Contribution analysis for each of the processes involved in “Banana at-the-foreign-port” for years 2019 and 2020.

Figure 4 shows the percentage contributions of inputs and outputs to each environmental impact category for the landed banana.

The flows involved in this stage were grouped by type (i.e., “Banana at-the-packaging-stage-gate”, electricity consumption in the additional stage, electricity at the Port of Guayaquil, fuel use in the port machinery, road transport from farm to port, maritime transport). The product flow of “Banana at-the-packaging-stage-gate” is a high contributor in the impact categories GWP100 (Rotterdam 56%, San Diego 66%), FDP (Rotterdam 38% and 42%, San Diego 48% and 53%), FEP (Rotterdam 78% and 80%, San Diego 83% and 84%), MEP (Rotterdam 78% and 74%, San Diego 85% and 83%), ODPinf (Rotterdam 22% and 25%, San Diego 30% and 33%), PMFP (Rotterdam 25% and 31%, San Diego 37% and 43%), POFP (Rotterdam 13% and 15%, San Diego 21% and 23%), and TAP100 (Rotterdam 38% and 47%, San Diego 52% and 61%) for the years 2019 and 2020, respectively.

The maritime transport is the second highest contributor in the categories FDP (Rotterdam 47 and 45%, San Diego 32 and 30%), ODPinf (Rotterdam 53 and 51%, San Diego 37 and 36%), PMFP (Rotterdam 65 and 63%, San Diego 53 and 48%), POFP (Rotterdam 78 and 77%, San Diego 65 and 64%), and TAP100 (Rotterdam 57 and 49%, San Diego 41 and 34%). Previous studies have shown that maritime transport accounted for 18% in a study on destination in Spain [17] and between 27% and 67% in a study on destination in Germany [12]. The latter study includes a best-case scenario and a worst-case scenario. In the best-case scenario, the banana travels in refrigerated container ships that transport other content when they return, thus only counting the kilometers traveled in one direction of the journey. In the worst-case scenario, the bananas travel in small refrigerated boats and the boats return empty, counting the kilometers of a round trip.

This difference is caused by the distance traveled by sea, requiring greater fuel consumption. This affects the processes of transport and cooling and their emissions.

Table 6, Table 7 and Table 8 shows the contributors to each impact category indicator results, in the period studied, for 1 ton “Banana at-the-port-of-destination”.

The product flow “Banana at-the-packaging-stage-gate” is a high contributor in the impact categories GWP100, FDP, FEP, MEP, ODPinf, PMFP, POFP, and TAP100 for the years 2019 and 2020.

Maritime transport is the second highest contributor in the GWP100 category, mostly associated with the emissions associated with this transport.

#### 3.2.4. Comparison with Carbon Footprint Studies of Banana in the Literature

**Figure 5:**
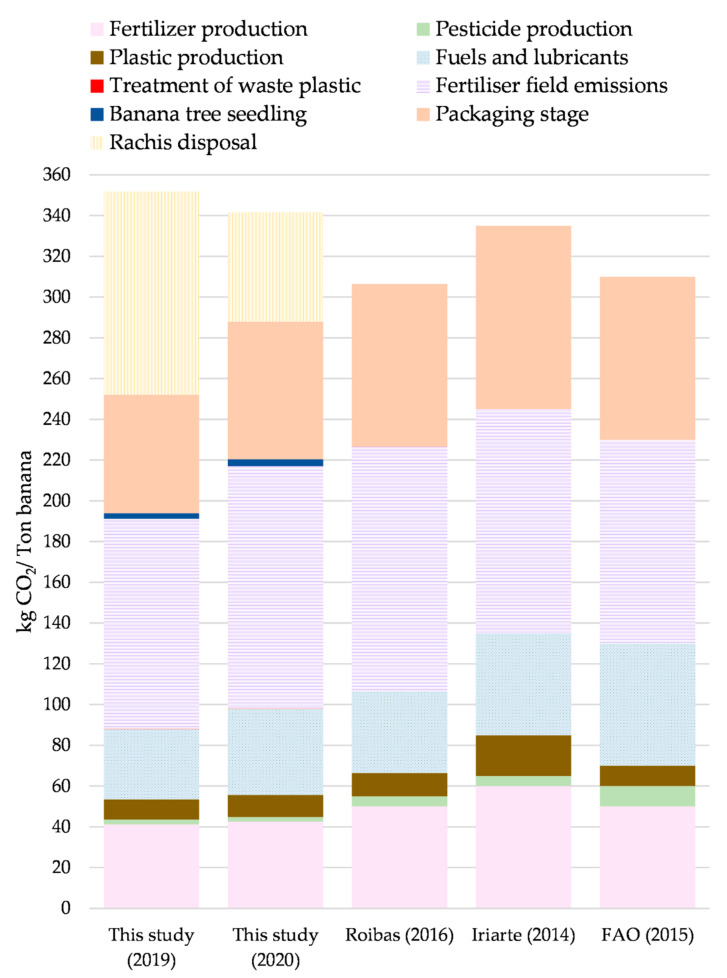
Comparison with carbon footprints of other studies [11,16,48], at “Banana packaging-stage-gate”.

Figure 5 shows the results of the two years in this study and of three reviewed studies as a comparative basis [12,17,49]. The carbon footprints, ranked from highest to lowest, are: this study (2019), this study (2020), Iriarte et al., (2014) [12], FAO (2016) [50], and Roibas et al., (2016) [17].

The impact caused by the fertilizer field emissions’ category stands out as the greatest contribution in the five cases, resulting in 103 and 119 kg CO_2_-Eq/Ton banana for the years 2019 and 2020, respectively, in this study. These results are similar to those obtained by Roibas [17], who obtained a contribution of 120 kg CO_2_-Eq/Ton banana.

For the packaging stage, the results of the carbon footprint of this study were 58 and 68 kg CO_2_-Eq/Ton banana for the years 2019 and 2020, respectively. These results are lower in relation to Roibas [17], FAO [51], and Iriarte [12]. The highest contribution to the carbon footprint of this study for this stage comes from the emissions from the production of cardboard, with the results of 49 and 56 kg CO_2_-Eq/Ton banana for the years 2019 and 2020, respectively.

The emissions from pesticide production contribute 2 kg CO_2_-Eq/Ton banana in this study; lower than the contribution in FAO [51] of 10 kg CO_2_-Eq/Ton banana and in Roibas [17] and Iriarte [12] of 5 kg CO_2_-Eq/Ton banana in both cases. The amount and type of pesticide use is different among all the studies. The farm where this study was developed was in the process of reducing the use of pesticides, switching to organic controls such as phytosanitary defoliation.

There is an important difference between this study and the previous studies, in particular regarding the inclusion of rachis management. In this study, emissions of 99 and 54 kg CO_2_-Eq/Ton banana were obtained for 2019 and 2020, respectively. The difference between the two years, as mentioned by the farm staff, is that these residues began to be registered in 2018 and since then they have tried to reduce the number of rejected bunches. In Roibas [17], the emissions related to agricultural residues dumped on the plantation and the nitrogen content of the residues were 1.9% [50]. FAO [51] includes emissions associated with organic waste dumped on the plantation. Using the IPCC guidelines for tropical soils, these emissions are not segregated. Iriarte [12] does not specify the inclusion of this type of flow. In this study, in the base case RM0, the management of the rachis has been modeled, establishing the worst possible situation after discarding in the field, including the effect of the decomposition in an open dump and the field emissions associated with the N_2_O volatilization.

### 3.3. Recommendations for Improvement

Good farming practices were performed and acknowledged, such as using fertilizers that positively affect soil fertility. However, their production and the emissions due to their application are the main contributors to the release of emissions. Thus, a plan should be developed to reduce the amount of high-nitrogen-content fertilizers [52,53]. Precision farming is a key strategy for the mitigation of impacts from agriculture. For the climate change category, there is the opportunity to optimize the application of ammonium nitrate and urea and to stimulate the application of organic fertilizers (e.g., compost) instead of mineral fertilizers.

The use of fossil fuels to produce energy resulted in one of the main contributions to almost all the impact categories in the banana growing phase. Thus, it is advised to use other renewable energy sources, considering the accessibility of these energy sources in each country, the high initial capital costs, and the economic benefits [27,54]

The banana packaging stage showed the smallest contribution along the whole cycle, and the main contributors were the use of cardboard for packaging and the disposal of the rachis. There are some alternatives for the use of the rachis, such as the production of bioethanol and the production of leachate of the rachis to be used as nutrition for the fields, mainly for the potassium content and smaller quantities of N, Fe, Mn, Na, and Cu., as studied by [55].

Residual banana aerial biomass such as rachis, leaves, and pseudostems could be used in a circular bioeconomy as described in [56]. Circular alternatives that show great potential are being used, such as the production of biopolymers, active carbon, and biofuels [57,58]. In addition, rachis could be used as a source of biomass through the fermentation of the solid state in conditioned soils [59] or used to obtain enzymes from the biomass in a biological base through the use of microorganisms, as seen in [60]. In addition, other domestic uses of rachis could be explored (e.g., to be used to tie or mark livestock, feed for livestock, composting) as shown by [61].

For the case of cardboard for packaging, strategies to reduce the amount of packaging should be investigated. Furthermore, packaging produced from biomass residues of the same value chain can be studied [56].

As with any other transport, decarbonization is also needed in maritime transportation. This would result in mitigating the impact of products that are sent overseas.

### 3.4. Limitations and Further Research

The main limitation of the study is that only one farm was used as the study site. Other limitations are impact categories that were not analyzed, such as metal depletion, ecotoxicity, agricultural land occupation, urban land occupation, water depletion, ionizing radiation, and natural land transformation. These impact categories require further investigation, as in the case of water depletion, requiring information on elemental flows of groundwater, lake water, river water, and water vapor.

Further study is required to describe the actual destiny of the rachis (and other residual biomass) during or after the growing and packaging stages.

## 4. Conclusions

The sustainability of banana in Ecuador is of great interest as it represents an important source of income for the country. This study has explored the carbon footprint and other impact categories not explored before in previous studies such as eutrophication, ozone depletion, photochemical oxidant, particulate matter, and terrestrial acidification.

The carbon footprint of Ecuadorian bananas delivered in Rotterdam (Netherlands) and San Diego (United States) was estimated to range between 615.41–625.44 kg CO_2_-Eq/Ton banana and between 520.37–530.40 CO_2_-Eq/Ton banana, respectively. The highest contribution came from the “Banana Growing Stage” (193.94–220.37 kg CO_2_-Eq/Ton banana). The results for the “Banana at-the-foreign-port” change depended on the distance to the destination port.

Emissions from the application of nitrogenous fertilizers during the “Banana Growing Stage” represented more than 50% of the GWP100, MEP, PMFP, and TAP100 impact categories. The use of fuels and lubricants was the second highest contributor, contributing more than 35% of the impact categories FDP, FEP, ODPinf, and POFP. Other products that contributed significantly to the carbon footprint were: using cardboard for packaging and maritime transport. Reducing the use of fertilizers with a high nitrogen content or alternatives is a key strategy to mitigate the impact.

The current study evaluates the Ecuadorian banana, mainly using primary data from a conventional farm where the type of fruit belongs to the genus Musa AAA “Banano Cavendish”. However, this is not the only specie that is currently produced in the country, where, for export purposes, AAB “Platano Verde” and AA ”Baby Banana” species are also produced in addition to other subspecies that change the production location depending on the environmental, soil, and geophysical variables of each region [62]. It is recommended to increase the amount of existing data related to other species and to evaluate the environmental performances of more farms.

## Figures and Tables

**Table 1:** Overview of life cycle assessment studies of banana reported in peer-reviewed journals.

Reference	System	Description and Boundaries	Geographic Limits	Functional Unit	Impact Categories
[20]	Banana production and supply to the end user in Turkey	Three scenarios were evaluated: conventional non-heated water; heating irrigation water with natural gas; heating irrigation water by using biogas from anaerobic digestion of banana stem residues. The system boundaries are cradle-to-grave.	Turkey, Anamur City, and the three big cities of Turkey (Ankara, Istanbul, Izmir). From agricultural production to delivery to a European port of destination	Total 2 ton of banana	Global warming potential, acidification potential, eutrophication potential, photochemical oxidants creation potential, ozone layer depletion potential, human toxicity potential
[21]	Food production in Hawaii	Assess the local (within Hawaii), distant (elsewhere), and global (both in Hawaii and elsewhere) environmental impacts of increased food production. The system boundaries are cradle-to-grave.	Islands Oahu, Maui, Kauai, and Hawaii	Total 1 kg of banana	Climate change, marine eutrophication, land use, water resource depletion
[13]	Two varieties of banana produced in Brazil: Cavendish and Prata	Determine environmental indicators for two varieties of banana produced in Brazil—Cavendish and Prata—in order to promote these products among consumers. The boundaries are cradle-to-gate.	Brazil: Ribeira Valley in Sao Paulo State and north of Minas Gerais	Total 1 kg of banana	Global warming potential, primary energy demand, abiotic depletion, eutrophication potential, acidification potential, land use, total freshwater use, blue water use, terrestrial ecotoxicity potential, human toxicity potential
[14]	Initiate and promote LCA in Uganda with the overall objective of promoting life cycle thinking to improve the competitiveness of agricultural products regionally and internationally	Evaluate and quantify the environmental impacts of the life cycle of selected products. The system boundaries are door-to-door based on processes in accordance with ISO 14040.	Uganda: Lubanja Village, Mityana District; Kangulumira, Kayunga District	Total 1 kg of banana	Carbon footprint, ecological toxicity, human toxicity, photochemical oxidation, abiotic depletion
[17]	Banana production in Ecuador direct to a European country	Life cycle evaluation of 17 Ecuadorian plantations (9 organic and 8 conventional, classified by size). The system boundaries are cradle-to-gate.	From Ecuador to Spain	Total 1 kg of banana	Water footprint, carbon footprint
[12]	Ecuadorian export premium banana production	Inventory of GHG emissions from Ecuadorian agricultural products using the concept of carbon footprint. The system boundaries are from agricultural production to delivery to a European port of destination (cradle-to-grave).	From Ecuador to Germany	Total 1 kg of banana	Carbon footprint
[18]	Project on bananas and climate change in Ecuador, as part of the FAO Multidonor Mechanism (FMM) program	Evaluation of the impacts of climate change on bananas in Ecuador. The study covers the entire supply chain, from production to final consumption.	From Ecuador to Madrid, Spain and port of Rotterdam, Holland	Total 1 kg of banana	Carbon footprint
[15]	Banana produced in two plantations in Costa Rica	Study the greenhouse gas emissions of bananas from cradle to retail and cradle to grave.	From Costa Rica to Oslo, Norway	Total 1 kg of banana	Carbon footprint
[16]	Fruit and vegetable production, including seedlings, use of machinery, greenhouse fuels, irrigation, fertilizers, pesticides, storage, and transport within Switzerland	Environmental assessment of an assortment of 34 fruits and vegetables from a large Swiss retailer	From 29 different countries to Switzerland	Total 1 kg of product	Climate change, water stress, human toxicity, eutrophication, acidification, soil fertility degradation, landscape changes.
[19]	Development of methods for analyzing product carbon footprint and life cycle assessment	Study on the development of methods for analyzing product carbon footprint (PCF) and life cycle assessment (LCA)	From Ecuador to east coast of USA or northern Europe	Total 1 kg of banana	Eutrophication, terrestrial eco-toxicity, sediment ecotoxicity, aquatic ecotoxicity, human toxicity

**Table 2:** Rachis management scenarios.

Scenario	Description
RM0	Base case: treatment in an open dump + N_2_O and NO_x_ emissions calculated based on IPCC
RM1	N_2_O and NO_x_ emissions calculated based on IPCC
RM2	No rachis treatment (no burden associated with this flow)

**Table 3:** Impact category indicator results for 1 ton of “Banana at-the-farm-gate” for years 2019 and 2020.

	GWP100		FDP		FEP		MEP		ODPinf		PMFP		POFP		TAP100	
	(kg CO_2_−Eq)		(kg Oil-Eq)		(kg P-Eq)		(kg N-Eq)		(kg CFC-11-Eq)		(kg PM_10_-Eq)		(kg NMVOC-Eq)		(kg SO_2_-Eq)	
	2019	2020	2019	2020	2019	2020	2019	2020	2019	2020	2019	2020	2019	2020	2019	2020
Fertilizer production	4.12 × 10^1^	4.25 × 10^1^	1.15 × 10^1^	1.39 × 10^1^	3.40 × 10^−3^	3.78 × 10^−3^	1.18 × 10^−2^	1.50 × 10^−2^	3.31 × 10^−6^	3.99 × 10^−6^	5.60 × 10^−2^	6.28 × 10^−2^	9.70 × 10^−2^	1.07 × 10^−1^	1.88 × 10^−1^	2.08 × 10^−1^
Pesticide production	2.37	2.27	1.14	1.19	1.59 × 10^−3^	1.51 × 10^−3^	1.28 × 10^−2^	9.18 × 10^−3^	1.01 × 10^−6^	5.22 × 10^−7^	6.26 × 10^−3^	6.04 × 10^−3^	8.98 × 10^−3^	8.52 × 10^−3^	1.92 × 10^−2^	1.81 × 10^−2^
Plastic production	9.92	1.08 × 10^1^	5.75	6.47	1.15 × 10^−3^	1.22 × 10^−3^	1.30 × 10^−3^	1.41 × 10^−3^	1.63 × 10^−7^	1.68 × 10^−7^	1.25 × 10^−2^	1.35 × 10^−2^	4.63 × 10^−2^	4.47 × 10^−2^	3.68 × 10^−2^	3.89 × 10^−2^
Fuels and lubricants	3.45 × 10^1^	4.24 × 10^1^	1.15 × 10^1^	1.41× 10^1^	4.45 × 10^−3^	5.52 × 10^−3^	7.84 × 10^−3^	9.63 × 10^−3^	5.92 × 10^−6^	7.27 × 10^−6^	7.14 × 10^−2^	8.76 × 10^−2^	2.38 × 10^−1^	2.88 × 10^−1^	1.58 × 10^−1^	1.95 × 10^−1^
Treatment of waste plastic	1.32 × 10^−1^	9.62 × 10^−2^					1.40 × 10^−3^	1.02 × 10^−3^			1.24 × 10^−7^	9.04 × 10^−8^	4.58 × 10^−5^	3.33 × 10^−5^	6.11 × 10^−7^	4.44 × 10^−7^
Fertilizer fieldemissions	1.03 × 10^2^	1.19 × 10^2^					6.60 × 10^−2^	1.01 × 10^−1^			2.36 × 10^−1^	3.58 × 10^−1^	7.26 × 10^−2^	8.39 × 10^−2^	1.72	2.65
Banana tree seedling	2.71	3.40	6.49× 10^−1^	8.13 × 10^−1^	1.29 × 10^−3^	1.61 × 10^−3^	9.70 × 10^−4^	1.21 × 10^−3^	1.95 × 10^−7^	2.44 × 10^−7^	7.38 × 10^−3^	9.24 × 10^−3^	9.69 × 10^−3^	1.21 × 10^−2^	1.34 × 10^−2^	1.68 × 10^−2^
**Total**	1.94 × 10^2^	2.20 × 10^2^	3.06 × 10^1^	3.66 × 10^1^	1.19 × 10^−2^	1.36 × 10^−2^	1.02 × 10^−1^	1.38 × 10^−1^	1.06 × 10^−5^	1.22 × 10^−5^	3.89 × 10^−1^	5.37 × 10^−1^	4.72 × 10^−1^	5.44 × 10^−1^	2.14	3.13

**Table 4:** Impact category indicator results for 1 ton of “Banana at-the-packaging-stage-gate” for years 2019 and 2020.

	GWP100		FDP		FEP		MEP		ODPinf		PMFP		POFP		TAP100	
	(kg CO_2_−Eq)		(kg Oil-Eq)		(kg P-Eq)		(kg N-Eq)		(kg CFC-11-Eq)		(kg PM_10_-Eq)		(kg NMVOC-Eq)		(kg SO_2_-Eq)	
	2019	2020	2019	2020	2019	2020	2019	2020	2019	2020	2019	2020	2019	2020	2019	2020
Banana at-the-farm-gate	1.94 × 10^2^	2,20 × 10^2^	3.06 × 10^1^	3.66 × 10^1^	1.19 × 10^−2^	1.36 × 10^−2^	1.02 × 10^−1^	1.38 × 10^−1^	1.06 × 10^−5^	1.22 × 10^−5^	3.89 × 10^−1^	5.37 × 10^−1^	4.72 × 10^−1^	5.44 × 10^−1^	2.14	3.13
Cardboard packaging	4.92 × 10^1^	5.65 × 10^1^	1.60 × 10^1^	1.84 × 10^1^	1.97 × 10^−2^	2.27 × 10^−2^	2.77 × 10^−2^	3.19 × 10^−2^	3.88 × 10^−6^	4.56 × 10^−6^	1.15 × 10^−1^	1.32 × 10^−1^	1.46 × 10^−1^	1.68 × 10^−1^	1.86 × 10^−1^	2.13 × 10^−1^
Plastic packaging	5.87	6.75	4.24	4.86	1.59 × 10^−3^	1.82 × 10^−3^	1.21 × 10^−3^	1.39 × 10^−3^	1.57 × 10^−7^	1.86 × 10^−7^	9.28 × 10^−3^	1.07 × 10^−2^	2.49 × 10^−2^	2.86 × 10^−2^	1.99 × 10^−2^	2.29 × 10^−2^
Electricity	2.33	2.43	7.81 × 10^−1^	8.16 × 10^−1^	2.37 × 10^−5^	2.48 × 10^−5^	9.00 × 10^−4^	9.40 × 10^−4^	3.39 × 10^−7^	3.54 × 10^−7^	7.44 × 10^−3^	7.77 × 10^−3^	1.53 × 10^−2^	1.59 × 10^−2^	2.23 × 10^−2^	2.33 × 10^−2^
Tap water	1.95 × 10^−1^	2.15 × 10^−1^	7.12 × 10^−2^	7.85 × 10^−2^	3.73 × 10^−5^	4.12 × 10^−5^	4.60 × 10^−5^	5.10 × 10^−5^	1.48 × 10^−8^	1.64 × 10^−8^	3.90 × 10^−4^	4.30 × 10^−4^	6.10 × 10^−4^	6.80 × 10^−4^	6.20 × 10^−4^	6.80 × 10^−4^
Cleaning agent production	5.69 × 10^−1^	1.71	2.37 × 10^−1^	1.15	1.41 × 10^−4^	7.67 × 10^−4^	9.65 × 10^−4^	1.37 × 10^−3^	4.60 × 10^−8^	1.52 × 10^−7^	1.96 × 10^−3^	1.58 × 10^−2^	2.20 × 10^−3^	1.66 × 10^−2^	6.75 × 10^−3^	6.67 × 10^−2^
Rachis disposal	9.96 × 10^1^	5.37 × 10^1^			3.59 × 10^−3^	2.45 × 10^−3^	4.73 × 10^−1^	3.23 × 10^−1^			1.04 × 10^−2^	4.87 × 10^−3^	6.20 × 10^−2^	3.22 × 10^−2^	2.65 × 10^−2^	1.24 × 10^−2^
**Total**	3.52 × 10^2^	3.42 × 10^2^	5.19 × 10^1^	6.18 × 10^1^	3.70 × 10^−2^	4.14 × 10^−2^	6.06 × 10^−1^	4.97 × 10^−1^	1.50 × 10^−5^	1.75 × 10^−5^	5.34 × 10^−1^	7.09 × 10^−1^	7.24 × 10^−1^	8.06 × 10^−1^	2.40	3.46

**Table 5:** Impact category indicator results for 1 ton “Banana at-the-packaging-stage-gate” for the three rachis management scenarios.

Impact Category		2019			2020		
Name	Unit	RM0	RM1	RM2	RM0	RM1	RM2
GWP100	kg CO_2_-Eq	361.9498	329.1613	262.3156	353.1016	330.6849	299.4056
FDP	kg oil-Eq	53.4987	53.4987	53.4987	63.7866	63.7866	63.7866
FEP	kg P-Eq	0.03764	0.03406	0.03406	0.04218	0.03973	0.03973
MEP	kg N-Eq	0.6116	0.1402	0.1384	0.5045	0.1822	0.1813
ODPinf	kg CFC-11-Eq	0.0000156	0.0000156	0.0000156	0.0000182	0.0000182	0.0000182
PMFP	kg PM_10_-Eq	0.55421	0.55419	0.54382	0.73733	0.73731	0.73246
POFP	kg NMVOC-Eq	0.74871	0.73384	0.68673	0.83534	0.82518	0.80314
TAP100	kg SO_2_-Eq	2.51381	2.51369	2.48731	3.62916	3.62907	3.61673

**Table 6:** Impact category indicator results for 1 ton of “Banana at-the-foreign-port” for years 2019 and 2020 for impact categories QWP100, FDP, and FEP.

	GWP100 (kg CO_2_-Eq)				FDP (kg oil-Eq)				FEP (kg P-Eq)			
	2019 Rotterdam	2019 San Diego	2020 Rotterdam	2020 San Diego	2019 Rotterdam	2019 San Diego	2020 Rotterdam	2020 San Diego	2019 Rotterdam	2019 San Diego	2020 Rotterdam	2020 San Diego
Banana at-the- packaging-stage-gate	3.52 × 10^2^	3.52 × 10^2^	3.42 × 10^2^	3.42 × 10^2^	5.19 × 10^1^	5.19 × 10^1^	6.18 × 10^1^	6.18 × 10^1^	3.70 × 10^−2^	3.70 × 10^−2^	4.14 × 10^−2^	4.14 × 10^−2^
Electricity collection center	2.03	2.03	2.03	2.03	6.83 × 10^−1^	6.83 × 10^−1^	6.84 × 10^−1^	6.84 × 10^−1^	2.07 × 10^−5^	2.07 × 10^−5^	2.08 × 10^−5^	2.08 × 10^−5^
Electricity at Guayaquil Port	3.94 × 10^−1^	3.94 × 10^−1^	3.94 × 10^−1^	3.94 × 10^−1^	1.33 × 10^−1^	1.33 × 10^−1^	1.33 × 10^−1^	1.33 × 10^−1^	4.07 × 10^−6^	4.07 × 10^−6^	4.07 × 10^−6^	4.07 × 10^−6^
Fuel use in port machinery	2.05	2.05	2.05	2.05	6.90 × 10^−1^	6.90 × 10^−1^	6.90 × 10^−1^	6.90 × 10^−1^	5.84 × 10^−5^	5.84 × 10^−5^	5.84 × 10^−5^	5.84 × 10^−5^
Road transport farm to port	6.39 × 10^1^	6.39 × 10^1^	6.39 × 10^1^	6.39 × 10^1^	1.91 × 10^1^	1.91 × 10^1^	1.91 × 10^1^	1.91 × 10^1^	4.24 × 10^−3^	4.24 × 10^−3^	4.24 × 10^−3^	4.24 × 10^−3^
Maritime transport	2.05 × 10^2^	1.10 × 10^2^	2.05 × 10^2^	1.10 × 10^2^	6.55 × 10^1^	3.52 × 10^1^	6.57 × 10^1^	3.52 × 10^1^	6.02 × 10^−3^	3.22 × 10^−3^	6.02 × 10^−3^	3.22 × 10^−3^
**Total**	6.25 × 10^2^	5.30 × 10^2^	6.15 × 10^2^	5.20 × 10^2^	1.38 × 10^2^	1.08 × 10^2^	1.48 × 10^2^	1.18 × 10^2^	4.73 × 10^−2^	4.45 × 10^−2^	5.17 × 10^−2^	4.89 × 10^−2^

GWP100—climate change; FDP—fossil depletion; FEP—freshwater eutrophication

**Table 7:** Impact category indicator results for 1 ton of “Banana at-the-foreign-port” for years 2019 and 2020 for impact categories MEP, ODPinf, and PMFP.

	MEP (kg N−Eq)				ODPinf (kg CFC-11-Eq)			PMFP (kg PM_10_-Eq)				
	2019 Rotterdam	2019 San Diego	2020 Rotterdam	2020 San Diego	2019 Rotterdam	2019 San Diego	2020 Rotterdam	2020 San Diego	2019 Rotterdam	2019 San Diego	2020 Rotterdam	2020 San Diego
Banana at-the- packaging-stage-gate	6.06 × 10^−1^	6.06 × 10^−1^	4.97 × 10^−1^	4.97 × 10^−1^	1.50 × 10^−5^	1.50 × 10^−5^	1.75 × 10^−5^	1.75 × 10^−5^	5.34 × 10^−1^	5.34 × 10^−1^	7.09 × 10^−1^	7.09 × 10^−1^
Electricity collection center	7.90 × 10^−4^	7.90 × 10^−4^	7.90 × 10^−4^	7.90 × 10^−4^	2.97 × 10^−7^	2.97 × 10^−7^	2.97 × 10^−7^	2.97 × 10^−7^	6.51 × 10^−3^	6.51 × 10^−3^	6.51 × 10^−3^	6.51 × 10^−3^
Electricity at Guayaquil Port	1.50 × 10^−4^	1.50 × 10^−4^	1.50 × 10^−4^	1.50 × 10^−4^	5.75 × 10^−8^	5.75 × 10^−8^	5.75 × 10^−8^	5.75 × 10^−8^	1.26 × 10^−3^	1.26 × 10^−3^	1.26 × 10^−3^	1.26 × 10^−3^
Fuel use in port machinery	9.90 × 10^−4^	9.90 × 10^−4^	9.90 × 10^−4^	9.90 × 10^−4^	3.55 × 10^−7^	3.55 × 10^−7^	3.55 × 10^−7^	3.55 × 10^−7^	8.52 × 10^−3^	8.52 × 10^−3^	8.52 × 10^−3^	8.52 × 10^−3^
Road transport farm to port	1.62 × 10^−2^	1.62 × 10^−2^	1.62 × 10^−2^	1.62 × 10^−2^	1.62 × 10^−5^	1.62 × 10^−5^	1.62 × 10^−5^	1.62 × 10^−5^	1.28 × 10^−1^	1.28 × 10^−1^	1.28 × 10^−1^	1.28 × 10^−1^
Maritime transport	1.54 × 10^−1^	8.27 × 10^−2^	1.54 × 10^−1^	8.27 × 10^−2^	3.58 × 10^−5^	1.92 × 10^−5^	3.58 × 10^−5^	1.92 × 10^−5^	1.45	7.77 × 10^−1^	1.45	7.77 × 10^−1^
**Total**	7.78 × 10^−1^	7.07 × 10^−1^	6.69 × 10^−1^	5.98 × 10^−1^	6.77 × 10^−5^	5.11 × 10^−5^	7.02 × 10^−5^	5.36 × 10^−5^	2.12	1.46	2.30	1,63

MEP—marine eutrophication; ODPinf—ozone depletion; PMFP—particulate matter formation

**Table 8:** Impact category indicator results for 1 ton of “Banana at-the-foreign-port” for years 2019 and 2020 by impact categories POFP and TAP100.

	POFP (kg NMVOC-Eq)				TAP100 (kg SO_2_-Eq)			
	2019 Rotterdam	2019 San Diego	2020 Rotterdam	2020 San Diego	2019 Rotterdam	2019 San Diego	2020 Rotterdam	2020 San Diego
Banana at-the- packaging-stage-gate	7.4 × 10^−1^	7.24 × 10^−1^	8.06 × 10^−1^	8.06 × 10^−1^	2.40	2.40	3.46	3.46
Electricity collection center	1.34 × 10^−2^	1.34 × 10^−2^	1.34 × 10^−2^	1.34 × 10^−2^	1.95 × 10^−2^	1.95 × 10^−2^	1.95 × 10^−2^	1.95 × 10^−2^
Electricity at Guayaquil Port	2.59 × 10^−3^	2.59 × 10^−3^	2.59 × 10^−3^	2.59 × 10^−3^	3.78 × 10^−3^	3.78 × 10^−3^	3.78 × 10^−3^	3.78 × 10^−3^
Fuel use in port machinery	2.90 × 10^−2^	2.90 × 10^−2^	2.90 × 10^−2^	2.90 × 10^−2^	1.64 × 10^−2^	1.64 × 10^−2^	1.64 × 10^−2^	1.64 × 10^−2^
Road transport farm to port	3.99 × 10^−1^	3.99 × 10^−1^	3.99 × 10^−1^	3.99 × 10^−1^	2.67 × 10^−1^	2.67 × 10^−1^	2.68 × 10^−1^	2.68 × 10^−1^
Maritime transport	4.15	2.23	4.15	2.23	3.60	1.93	3.60	1.93
**Total**	5.32	3.40	5.40	3.48	6.30	4.64	7.37	5.70

POFP—photochemical oxidant formation; TAP100—terrestrial acidification

## Data Availability

Not applicable.

## Supplementary Materials

The following supporting information can be downloaded at: https://www.mdpi.com/article/10.3390/foods11203288/s1, Table S1 Inventory of inputs and outputs per functional unit (1Ton) Banana at-the-farm-gate, Table S2 Inventory of inputs and outputs per functional unit (1Ton) Banana at-the-packaging-stage-gate.
