# Supplementary material for: The Environmental Profile of Ecuadorian Export Banana: A Life Cycle Assessment
Source: Foods. 2022 Oct 20;11(20):3288. doi: 10.3390/foods11203288 (PMC9601378; doi:10.3390/foods11203288)
Supplement: Supplementary file 1 [file foods-11-03288-s001.zip › foods-1869822-supplementary.pdf]

Supplementary Information

S1. Life cycle inventory

S1.1 Banana at-the-farm-gate

Table S1. Inventory of inputs and outputs per functional unit (1Ton) Banana at-the-farm-gate.

Inputs					
**Flow**	**Amount**		**Unit**	**Flow type**	**Life cycle inventory source**
	**2019**	**2020**			
Algae (Laminaria), dried, consumption mix	1.72E-02	1.73E-02	kg	Product flow	Ecoinvent [34] Algae (Laminaria), dried, consumption mix - FR
Alkyl sulphate (C12-14)	1.24E-03	3.11E-05	kg	Product flow	Ecoinvent [34] market for alkyl sulphate (C12-14) ǀ alkyl sulphate (C12-14) ǀ APOS, U - GLO
Aminopyridine	1.18E-02	8.30E-03	kg	Product flow	Ecoinvent [34] market for aminopyridine ǀ aminopyridine ǀ APOS, U - RoW
Ammonium nitrate, as N	2.56E+00	1.40E+00	kg	Product flow	Ecoinvent [34] market for ammonium nitrate, as N ǀ ammonium nitrate, as N ǀ APOS, U - GLO
Ammonium sulfate, as N	9.85E-01	7.63E-01	kg	Product flow	Ecoinvent [34] market for ammonium sulfate, as N ǀ ammonium sulfate, as N ǀ APOS, U - GLO
Benzoic acid	1.06E-03	6.47E-02	kg	Product flow	Ecoinvent [34] market for benzoic acid ǀ benzoic acid ǀ APOS, U - RoW
Bipyridylium-compound	1.79E-02	6.68E-03	kg	Product flow	Ecoinvent [34] market for bipyridylium-compound ǀ bipyridylium-compound ǀ APOS, U - GLO
Butyrolactone	1.24E-03	1.57E-03	kg	Product flow	Ecoinvent [34] market for butyrolactone ǀ butyrolactone ǀ APOS, U - RoW

Calcium carbonate	4.12E-02	1.33E-02	kg	Product flow	Ecoinvent [34] market for calcium carbonate, precipitated ǀ calcium carbonate, precipitated ǀ APOS, U - RoW
Calcium nitrate	1.12E-04	7.56E-05	kg	Product flow	Ecoinvent [34] market for calcium nitrate ǀ calcium nitrate ǀ APOS, U - GLO
Chlorothalonil	1.22E-02	6.21E-03	kg	Product flow	Ecoinvent [34] market for chlorothalonil ǀ chlorothalonil ǀ APOS, U - GLO
Diammonium phosphate (DAP) (with 18% N, 46% P2O5)	1.80E+00	2.55E+00	kg	Product flow	Ecoinvent [34] Diammonium phosphate (18% N 46% P2O5), at plant (WFLDB 3.5) - RER
Dithiocarbamated-compound	1.33E-03	6.63E-03	kg	Product flow	Ecoinvent [34] market for dithiocarbamate-compound ǀ dithiocarbamate-compound ǀ APOS, U - GLO
Esters of versatic acid	2.13E-03	1.29E-01	kg	Product flow	Ecoinvent [34] market for esters of versatic acid ǀ esters of versatic acid ǀ APOS, U - RoW
Ethylene glycol	0.00E+00	2.00E-03	kg	Product flow	Ecoinvent [34] market for ethylene glycol ǀ ethylene glycol ǀ APOS, U - GLO
Fruit tree seedling, for planting	2.27E+00	2.84E+00	item	Product flow	Ecoinvent [34] market for fruit tree seedling, for planting ǀ fruit tree seedling, for planting ǀ APOS, U - GLO
Glyphosate	1.01E-02	9.76E-03	kg	Product flow	Ecoinvent [34] market for glyphosate ǀ glyphosate ǀ APOS, U - GLO
Limestone, crushed, for mill	1.49E+00	4.43E-01	kg	Product flow	Ecoinvent [34] market for limestone, crushed, for mill ǀ limestone, crushed, for mill ǀ APOS, U - RoW
Lubricant oil	5.37E-02	7.00E-02	kg	Product flow	Ecoinvent [34] market for lubricating oil ǀ lubricating oil ǀ APOS, U - RoW
Magnesium	1.77E-09	1.07E-09	kg	Product flow	Ecoinvent [34] market for magnesium ǀ magnesium ǀ APOS, U - GLO
Magnesium sulfate	3.15E-01	2.30E-01	kg	Product flow	Ecoinvent [34] market for magnesium sulfate ǀ magnesium sulfate ǀ APOS, U - GLO
Mancozeb	4.06E-02	3.92E-02	kg	Product flow	Ecoinvent [34] market for mancozeb ǀ mancozeb ǀ APOS, U - GLO

Nitrogen fertiliser, as N	1.20E-01	3.52E-01	kg	Product flow	Ecoinvent [34] market for nitrogen fertiliser, as N ǀ nitrogen fertiliser, as N ǀ APOS, U - GLO
Occupation, agriculture	2.42E+02	2.67E+02	m²*a	Elementary flow	-
Organic fertiliser, 3-2-3, granulate, packaged	2.28E+00	6.69E+00	kg	Product flow	Ecoinvent [34] Organic fertiliser, 3-2-3, granulate, packaged
Organophosphorus-compound, unspecified	6.64E-02	6.29E-02	kg	Product flow	Ecoinvent [34] market for organophosphorus-compound, unspecified ǀ organophosphorus-compound, unspecified ǀ APOS, U - GLO
Packaging film, low density polyethylene	1.18E+00	1.29E+00	kg	Product flow	Ecoinvent [34] market for packaging film, low density polyethylene - GLO
Pesticide, unspecified	1.43E-02	6.29E-03	kg	Product flow	Ecoinvent [34] market for pesticide, unspecified ǀ pesticide, unspecified ǀ APOS, U - GLO
Petrol, unleaded, burned in machinery	5.65E+00	5.49E+00	MJ	Product flow	Ecoinvent [34] market for petrol, unleaded, burned in machinery ǀ petrol, unleaded, burned in machinery ǀ APOS, U - GLO
Petrol, unleaded, burned in machinery	1.77E+01	1.83E+01	MJ	Product flow	Ecoinvent [34] market for petrol, unleaded, burned in machinery ǀ petrol, unleaded, burned in machinery ǀ APOS, U – GLO
Phosphate fertiliser, as P2O5	2.79E-04	4.80E-04	kg	Product flow	Ecoinvent [34] market for phosphate fertiliser, as P2O5 ǀ phosphate fertiliser, as P2O5 ǀ APOS, U - GLO
Piperidine	2.36E-02	1.53E-02	kg	Product flow	Ecoinvent [34] market for piperidine ǀ piperidine ǀ APOS, U - GLO
Plastic cork stropper, PA, at plant	9.95E-01	6.23E-01	kg	Product flow	Ecoinvent [34] Bag, LDPE, at plant ǀ Plastic cork stopper, at plant - RER
Plastic film, PA, at plant	3.75E-01	4.20E-01	kg	Product flow	Ecoinvent [34] Plastic film, PA, at plant (ACYVIA) - RER
Plastic film, PA, at plant	1.30E-02	1.61E-02	kg	Product flow	Ecoinvent [34] Plastic film, PA, at plant (ACYVIA) - RER
Polypropylene, granulate	2.00E-01	7.47E-01	kg	Product flow	Ecoinvent [34] market for polypropylene, granulate ǀ polypropylene, granulate ǀ APOS, U - GLO

Polypropylene, granulate	1.55E-02	2.90E-02	kg	Product flow	Ecoinvent [34] market for polypropylene, granulate ǀ polypropylene, granulate ǀ APOS, U - GLO
Potassium chloride (with 60% K2O)	1.81E+01	1.79E+01	kg	Product flow	Ecoinvent [34] Potassium chloride (60% K2O), at plant (WFLDB 3.5) - RER
Potassium fertiliser, as K2O	1.91E-03	2.58E-03	kg	Product flow	Ecoinvent [34] market for potassium fertiliser, as K2O ǀ potassium fertiliser, as K2O ǀ APOS, U - GLO
Urea, as N	2.70E+00	4.77E+00	kg	Product flow	Ecoinvent [34] market for urea, as N ǀ urea, as N ǀ APOS, U - GLO
Water pump operation, diesel	3.50E+02	4.35E+02	MJ	Product flow	Ecoinvent [34] water pump operation, diesel ǀ water pump operation, diesel ǀ APOS, U - PE
Water, river	9.02E+01	1.25E+02	m^3	Elementary flow	-
		Outputs			
Flow	Amount		Unit	Flow type	Life cycle inventory source
	2019	2020			
Ammonia	6,87E-01	1,06E+00	kg	Elementary flow	-
Banana at the farm gate	1,00E+00	1,00E+00	Mg	**Product flow**	-
Carbon dioxide	4,98E+00	7,80E+00	kg	Elementary flow	-
Chlorothalonil	5,05E-04	2,57E-04	kg	Elementary flow	-
Chlorothalonil	9,46E-04	4,81E-04	kg	Elementary flow	-
Chlorothalonil	5,28E-07	2,68E-07	kg	Elementary flow	-

Dinitrogen monoxide	3,46E-01	3,99E-01	kg	Elementary flow	-
Diquat	1,83E-04	6,60E-05	kg	Elementary flow	-
Diquat	5,76E-03	2,08E-03	kg	Elementary flow	-
Diquat	1,53E-10	5,51E-11	kg	Elementary flow	-
Fenpropidin	2,35E-02	1,53E-02	kg	Elementary flow	-
Glufosinate-ammonium	1,28E-03	1,15E-03	kg	Elementary flow	-
Glufosinate-ammonium	1,78E-05	1,59E-05	kg	Elementary flow	-
Glufosinate-ammonium	2,94E-06	2,64E-06	kg	Elementary flow	-
Glyphosate	1,83E-04	1,77E-04	kg	Elementary flow	-
Glyphosate	3,13E-03	3,03E-03	kg	Elementary flow	-
Glyphosate	4,35E-06	4,21E-06	kg	Elementary flow	-
Imidacloprid	3,27E-05	3,26E-05	kg	Elementary flow	-
Imidacloprid	1,02E-03	1,02E-03	kg	Elementary flow	-
Imidacloprid	2,08E-07	2,08E-07	kg	Elementary flow	-
Mancozeb	7,28E-04	7,05E-04	kg	Elementary flow	-

Mancozeb	2,86E-14	2,77E-14	kg	Elementary flow	-
Mancozeb	4,90E-15	4,76E-15	kg	Elementary flow	-
Metiram	2,38E-05	1,19E-04	kg	Elementary flow	-
Metiram	1,16E-10	5,77E-10	kg	Elementary flow	-
Metiram	2,12E-11	1,05E-10	kg	Elementary flow	-
Nitrate	4,81E+01	5,73E+01	kg	Elementary flow	-
Nitrogen oxides	7,26E-02	8,39E-02	kg	Elementary flow	-
Paraquat	1,58E-04	6,14E-05	kg	Elementary flow	-
Paraquat	4,97E-03	1,93E-03	kg	Elementary flow	-
Paraquat	1,32E-10	5,00E-11	kg	Elementary flow	-
Phosphate	1,60E-04	2,20E-04	kg	Elementary flow	-
Pyrimethanil	2,30E-04	1,62E-04	kg	Elementary flow	-
Pyrimethanil	9,55E-07	6,71E-07	kg	Elementary flow	-
Pyrimethanil	1,33E-07	9,35E-08	kg	Elementary flow	-
Spiroxamine	1,22E-02	3,41E-03	kg	Elementary flow	-

Terbufos	5,57E-05	3,43E-05	kg	Elementary flow	-
Terbufos	3,29E-04	2,03E-04	kg	Elementary flow	-
Terbufos	5,41E-08	3,33E-08	kg	Elementary flow	-
Thiamethoxam	5,73E-05	3,48E-03	kg	Elementary flow	-
Thiamethoxam	1,03E-03	6,27E-02	kg	Elementary flow	-
Triflusulfuron-methyl	3,22E-06	1,42E-05	kg	Elementary flow	-
Triflusulfuron-methyl	2,36E-06	1,04E-05	kg	Elementary flow	-
Triflusulfuron-methyl	1,49E-08	6,54E-08	kg	Elementary flow	-
waste polyethylene	1,08E+00	7,85E-01	kg	Waste flow	Ecoinvent [34] treatment of waste polyethylene, open dump, dry infiltration class (100mm) ǀ waste polyethylene ǀ APOS, U - GLO

S1.2 Banana at-the-packaging-stage-gate

Table S2. Inventory of inputs and outputs per functional unit (1Ton) Banana at-the-packaging-stage-gate

Inputs					
Flow	Amount		Unit	Flow type	Life cycle inventory source
	2019	2020			
Alkylbenzene sulfonate, linear, petrochemical	8.11E-02	7.71E-02	kg	Product flow	Ecoinvent [34] market for alkylbenzene sulfonate, linear, petrochemical ǀ alkylbenzene sulfonate, linear, petrochemical ǀ APOS, U - GLO
Aluminium sulfate, powder	4.90E-02	4.76E-02	kg	Product flow	Ecoinvent [34] market for aluminium sulfate, powder ǀ aluminium sulfate, powder ǀ APOS, U - RoW
Ammonium chloride	5.71E-02	1.35E-02	Kg	Product flow	Ecoinvent [34] market for ammonium chloride ǀ ammonium chloride ǀ APOS, U - GLO
Banana at the farm gate	1.05E+00	1.05E+00	ton	Product flow	-
Benzimidazole-compound	1.24E-02	1.09E-02	kg	Product flow	Ecoinvent [34] market for benzimidazole-compound ǀ benzimidazole-compound ǀ APOS, U - GLO
Carton board box production	7.08E+01	8.12E+01	kg	Product flow	Ecoinvent [34] market for carton board box production, with gravure printing ǀ carton board box production, with gravure printing ǀ APOS, U - GLO
Chlorinated alkaline detergent, with gravure printing	1.68E-03	9.48E-03	kg	Product flow	Ecoinvent [34] Chlorinated alkaline detergent, for the meat industry, at plant - RER
Citric acid	1.56E-03	1.00E-03	kg	Product flow	Ecoinvent [34] market for citric acid ǀ citric acid ǀ APOS, U - GLO
Electricity, low voltage	1.59E+01	1.66E+01	kWh	Product flow	Ramirez, et al. [37] Electricity, at supply, 2018 mix - EC
Generic detergent-disinfectant	1.24E-02	4.17E-02	kg	Product flow	Ecoinvent [34] Generic detergent-disinfectant, at plant - RER

Hydrogen peroxide	4.55E-02	4.35E-03	kg	Product flow	Ecoinvent [34] market for hydrogen peroxide, without water, in 50% solution state ǀ hydrogen peroxide, without water, in 50% solution state ǀ APOS, U - RoW
Imidazole	4.13E-03	1.52E-03	kg	Product flow	Ecoinvent [34] market for imidazole ǀ imidazole ǀ APOS, U - GLO
Monochlorobenzene		2.62E-03	kg	Product flow	Ecoinvent [34] market for monochlorobenzene ǀ monochlorobenzene ǀ APOS, U - RoW
Non-ionic surfactant	2.57E-04	1.87E-02	kg	Product flow	Ecoinvent [34] market for non-ionic surfactant ǀ non-ionic surfactant ǀ APOS, U - GLO
Packaging film, low density polyethylene	2.00E+00	2.30E+00	kg	Product flow	Ecoinvent [34] market for packaging film, low density polyethylene ǀ packaging film, low density polyethylene ǀ APOS, U - GLO
Soybean oil, crude	2.41E-02	2.60E-02	kg	Product flow	Ecoinvent [34] market for soybean oil, crude ǀ soybean oil, crude ǀ APOS, U - GLO
Sulfuric acid	1.25E-01	9.26E+00	kg	Product flow	Ecoinvent [34] market for sulfuric acid ǀ sulfuric acid ǀ APOS, U - RoW
Tap water	4.04E+02	4.45E+02	kg	Product flow	Ecoinvent [34] market for tap water ǀ tap water ǀ APOS, U - PE
Vinyl acetate		1.10E-02	kg	Product flow	Ecoinvent [34] market for vinyl acetate ǀ vinyl acetate ǀ APOS, U - GLO

Outputs					
Flow	Amount		Unit	Flow type	Life cycle inventory source
	2019	2020			
Azoxystrobin		4,82E-05	kg	Elementary flow	-
Azoxystrobin		1,15E-03	kg	Elementary flow	-

Azoxystrobin		1,34E-07	kg	Elementary flow	-
Banana at the packaging stage gate	1,00E+00	1,00E+00	Mg	**Product flow**	-
Prochloraz	2,58E-04	1,93E-04	kg	Elementary flow	-
Prochloraz	8,92E-03	6,70E-03	kg	Elementary flow	-
Prochloraz	9,65E-07	7,24E-07	kg	Elementary flow	-
Rachis, after banana packaging	4,78E+02	3,27E+02	kg	Waste flow	(This study) Banana_Rachis disposal - EC

S2. Final disposition of the rachis at the “Banana Packaging Stage”

For describe the three scenarios of the final disposal of the rachis, table S3 shows the inventory analysis of the "Rachis, after banana packaging".

Table S3. Inventory of inputs and outputs per 1 kg of “Rachis, after banana packaging”, for the RM0 scenario.

Inputs					
Flow	Amount		Unit	Flow type	Life cycle inventory source
	2019	2020			
Rachis, after banana packaging	1	1	kg	Waste flow	(This study) Banana_Rachis disposal - EC
Outputs					
Flow	Amount		Unit	Flow type	Life cycle inventory source
	2019	2020			
Dinitrogen monoxide	4,69E-4	3,21E-4	kg	Elementary flow	
Nitrogen oxides	9,85E-5	6,74E-5	kg	Elementary flow	
Waste wood, untreated	1	1	kg	Waste flow	Ecoinvent [34] treatment of waste wood, untreated, open dump, moist infiltration class (300mm) | waste wood, untreated | APOS, U

Table S4. Inventory of inputs and outputs per 1 kg of “Rachis, after banana packaging”, for the RM1 scenario.

Inputs					
Flow	Amount		Unit	Flow type	Life cycle inventory source
	2019	2020			
Rachis, after banana packaging	1	1	kg	Waste flow	(This study) Banana_Rachis disposal - EC
Outputs					
Flow	Amount		Unit	Flow type	Life cycle inventory source
	2019	2020			
Dinitrogen monoxide	4,69E-4	3,21E-4	kg	Elementary flow	
Nitrogen oxides	9,85E-5	6,74E-5	kg	Elementary flow	

Table S5. Inventory of inputs and outputs per 1 kg of "Rachis, after banana packaging", for the RM2 scenario.

Inputs					
Flow	Amount		Unit	Flow type	Life cycle inventory source
	2019	2020			
Rachis, after banana packaging	1	1	kg	Waste flow	(This study) Banana_Rachis disposal - EC

The first scenario (RM0) considered all inputs and outputs, including nitrogen emissions from the spine (dinitrogen monoxide and nitrogen oxides) and treatment (wood residues, untreated). The second scenario (RM1) considers all the inputs and outputs, and the nitrogen emissions from the spine. The third scenario (RM2) only includes all inputs and outputs, not counting the rachis. In the column of considered scenarios, indicate the scenario where the flow was used.

S3. Uncertainty analysis

Figure S1 shows the results obtained in the uncertainty analysis, applying the Monte Carlo model. Performing 500 iterations, using OpenLCA software.

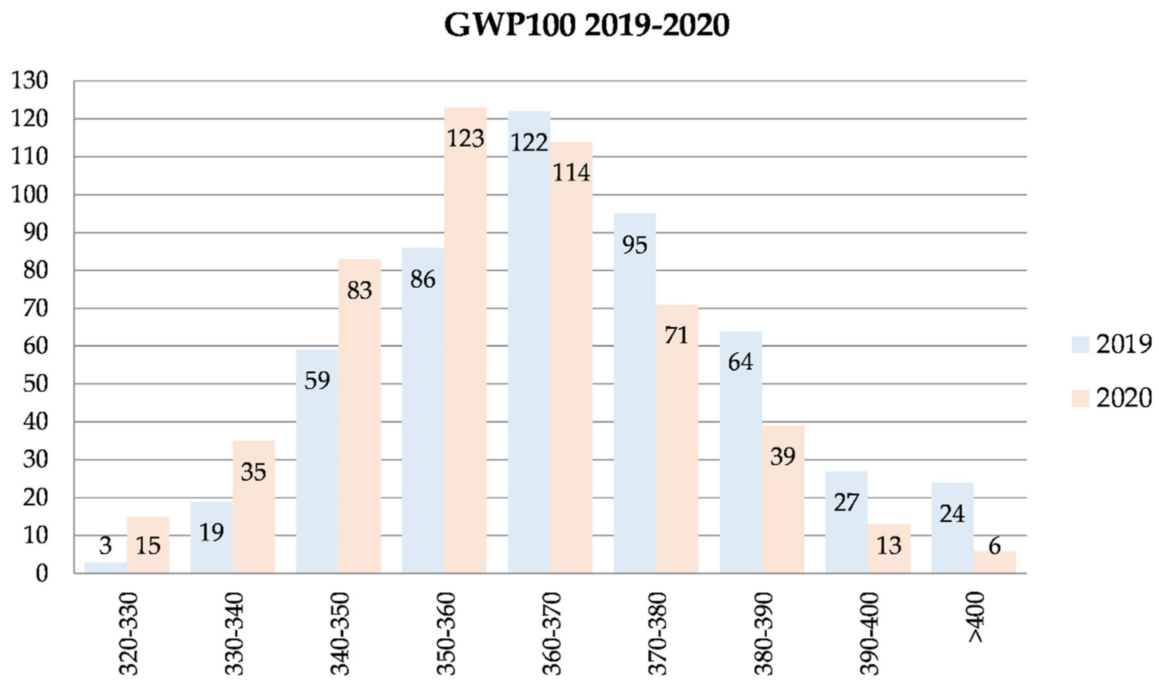

Figure S1. Histogram of the uncertainty analysis 2019-2020.

The uncertainty analysis was carried out for the Global Warming Potential (GWP100) category, at the "Banana Packaging Stage", for the RM0 scenario. The largest amount of iterations results are located in the range of 350-370 kg CO_2-Eq/Ton banana. In 2019, an average of 367,85 kg CO_2-Eq/Ton banana was obtained with a standard deviation of 17,77 kg CO_2-Eq/Ton banana. In 2020, an average of 360,17 kg CO_2-Eq/Ton banana was obtained with a standard deviation of 16,26 kg CO_2-Eq/Ton banana.

According to the results of this study in 2019 (352 kg CO_2-Eq/Ton banana), this is in the range of the uncertainty analysis (350.08-385.62 kg CO_2-Eq/Ton banana). For the year 2020 (342 kg CO_2-Eq/Ton banana), this is below the range of the uncertainty analysis (343.91-376.43 kg CO_2-Eq/Ton banana).

References

34. Ecoinvent The Ecoinvent Database. Available online: https://www.ecoinvent.org/database/database.html.

37. Ramirez, A.D.; Boero, A.; Rivela, B.; Melendres, A.M.; Espinoza, S.; Salas, D.A. Life Cycle Methods to Analyze the Environmental Sustainability of Electricity Generation in Ecuador: Is Decarbonization the Right Path? *Renew. Sustain. Energy Rev.* **2020**, *134*, 110373, doi:10.1016/j.rser.2020.110373.
